# Optimizing the Heat Treatment Method to Improve the Aging Response of Al-Fe-Ni-Sc-Zr Alloys

**DOI:** 10.3390/ma17081772

**Published:** 2024-04-12

**Authors:** Mingliang Wang, Zeyu Bian, Ailin Zhu, Yulong Cai, Dongdong Zhang, Yanlai Wu, Shuai Cui, Dong Chen, Haowei Wang

**Affiliations:** 1 State Key Laboratory of Metal Matrix Composites, Shanghai Jiao Tong University, Shanghai 200240, China; a.l.zhu@sjtu.edu.cn (A.Z.); chend@sjtu.edu.cn (D.C.); hwwang@sjtu.edu.cn (H.W.); 2 Institute of Alumics Materials, Shanghai Jiao Tong University (Anhui), Huaibei 235000, China; 3 Anhui Province Industrial Generic Technology Research Center for Alumics Materials, Huaibei Normal University, Huaibei 235000, China; 4 Innovation Academy for Microsatellites of Chinese Academy of Sciences, Shanghai 201203, China; caiyl@microsate.com (Y.C.); zhangdd@microsate.com (D.Z.); wuyl@microsate.com (Y.W.); cuis@microsate.com (S.C.)

**Keywords:** Al-Sc-Zr alloy, eutectic Al alloys, two-step aging, homogenization, hardness

## Abstract

This work has studied the co-addition of Sc and Zr elements into the Al-1.75wt%Fe-1.25wt%Ni eutectic alloy. The changes in the microstructure, electrical conductivity, and Vickers hardness of the Al-1.75wt%Fe-1.25wt%Ni-0.2wt%Sc-0.2wt%Zr alloy during heat treatment were studied. The results showed that two-step aging can effectively improve the aging response of the alloy over the single-step aging method. This was ascribed to the minimization of the diffusion difference between Sc and Zr elements. Furthermore, the homogenization treatment can also improve the aging response of the alloy by alleviating the uneven distribution of Sc and Zr. Nevertheless, the micro-alloyed elements exceeded the solid solubility limit in the Al-1.75wt%Fe-1.25wt%Ni-0.2wt%Sc-0.2wt%Zr alloy, and their strengthening effect has ever achieved the best prospect. Finally, both Sc and Zr contents were reduced simultaneously, and the aging response of the Al-1.75wt%Fe-1.25wt%Ni-0.15wt%Sc-0.1wt%Zr alloy was improved by optimized heat treatment. The underlying mechanisms for this alloy design and the corresponding microstructure–mechanical property relationship were analytically discussed.

## 1. Introduction

Since Al alloys have lower density and higher specific strength, they are widely applied in automobile and aerospace fields. A variety of heat-resistant Al alloys have been developed to meet the requirements for applications under high-temperature environments. As typical heat-resistant Al alloys, Al-Si- and Al-Cu-based alloys can still work at high temperatures (≤250 °C) for a long duration due to their good mechanical properties [1,2]. However, when these alloys are exposed to higher temperatures, the strengthening phases (i.e., Mg_2_Si and Al_2_Cu) should coarsen quickly and lose their reinforcing effect [3]. Moreover, the eutectic Si network inside the Al-Si alloy undergoes the spheroidization process, and fractures at higher temperatures occur, resulting in a sharp decline in mechanical properties [4,5]. The coarsening behaviors of both eutectic Si and the strengthening phases are closely related to the higher solubility or higher diffusion rate of the elements (i.e., Si or Cu) in the Al matrix. These factors make it difficult for Al-Si- and Al-Cu-based alloys to be applied at higher temperatures (300~400 °C).

It is a novel idea to use alloy elements (i.e., rare earth (Re) and transitional metal (TM) elements) with a slower diffusion rate to develop a new series of heat-resistant Al alloys. For example, Re elements (i.e., Y [6], Er [6,7], Yb [8], or Gd [8]) were used to develop novel heat-resistant Al-Cu-based alloys. These things considered, some eutectic systems with good casting properties (i.e., Al-Re (Re = La, Ce, Nd) and Al-TM (TM = Ni, Fe)) were proposed [9,10,11,12,13]. The Al-1.75wt%Fe-1.25wt%Ni eutectic alloy is this type of cast Al alloy, which has acceptable casting and mechanical performance. Koutsoukis et al. [14,15] reported that the Al-Fe-Ni eutectic alloy had similar fluidity and hot cracking properties to those of Al-Si alloys. Furthermore, the Al-Fe-Ni eutectic alloy showed improved mechanical properties over the Al-Si alloy in either ambient or higher temperatures [14]. In addition, both Fe and Ni elements have lower diffusion rates than those of Cu, Mg, and Si in an Al matrix [16], and the Al-Fe-Ni alloy has a much higher eutectic temperature (~650 °C) than the Al-Si alloy. This means that the Al-Fe-Ni alloy has higher thermal stability. Bian et al. [17,18] found that the needle-like eutectic Al_9_FeNi phase inside the Al-Fe-Ni alloy can retain its stability for a long time at 400 °C without coarsening. Therefore, the Al-Fe-Ni alloys should have great application prospects in heat-resistant Al alloys [19].

Precipitation strengthening is one of the most critical strengthening methods in Al alloys [20,21]. To develop more advanced heat-resistant Al alloys, it is necessary to explore the possibility of improving the Al matrix by precipitation strengthening. The Sc-containing Al alloys can precipitate nano-dispersed Al_3_Sc coherent with the matrix by the heat treatment process [22,23,24,25]. The induced nano Al_3_Sc particles have a significant reinforcing effect, and they can withstand higher temperatures (300 °C) without the coarsening phenomenon. When the pure Al contains 0.3wt%Sc, the tensile yield strength increases by 170 MPa at an ambient temperature, and the 300 °C creep properties of pure Al are greatly enhanced [26]. There are also many studies on introducing Sc into Al-Cu or Al-Si systems to improve their high-temperature performance. For instance, Chen and Sun [27] found that the peak-aged hardness of the Al-2.5wt%Cu-0.3wt%Sc alloy was significantly improved by 90% compared to that of the alloy without Sc.

In a severer condition, the Al_3_Sc phase inevitably coarsens and loses its reinforcing effect at ~400 °C [28,29,30]. Therefore, some micro-alloyed elements (Zr/Ti/Yb/Gd/Y) are co-incorporated with Sc to improve the high-temperature stability of the precipitated phase. For example, the L1_2_ Al_3_(Sc_x_Y_y_) precipitates can homogenously nucleate in an Al matrix and produce stabilized growth during annealing at 400 °C [31]. Nevertheless, Zr shows a superior function [32,33,34]. In the precipitates, Zr can partially replace the Sc position to form an Al_3_(Sc, Zr) core–shell structure [35,36,37,38]. The Sc element is mainly concentrated on the core, and the Zr element is mainly concentrated on the shell [39,40]. Since Zr has a much lower diffusion rate than that of Sc, this reduces the coarsening kinetics of the precipitate. Knipling et al. [33,41] reported the Al-Sc-Zr alloys kept microstructural stability for 100 h when they were exposed at ~400 °C.

In general, Al alloys (Al-Cu, etc. [42]) should undergo solid-solution and aging treatment to achieve the improved reinforcement effects. Similarly, Al alloys co-added with Sc and Zr elements should also be strengthened by the heat treatment strategy, and the detailed procedure has an intensive impact on Al alloy properties. Currently, the related heat treatment method is used in many studies. For example, isothermal aging (single-step aging) [32,43], two-step aging [24], or isochronal aging [33,41] can be used for Al-Sc-Zr alloys. In addition, there are several reports involved with the homogenization of the unevenly distributed Sc and Zr elements in an Al matrix [40,44]. Generally, these distinctive heat treatment methods should lead to diversified final performances.

This research studied both Sc and Zr micro-alloyed elements in the Al-Fe-Ni eutectic alloy to tune microstructural and mechanical properties. There was a previous work introducing the Sc element to explore changes in the mechanical properties of the eutectic Al-Fe-Ni alloy [45]. Therefore, this work was utilized to explore the effect of heat treatment methods on precipitation behavior with the Sc and Zr co-addition. Due to the uneven distribution of micro-alloyed elements in the eutectic structure, the heat treatment method is also different from that for pure Al. We investigated the aging and homogenization behaviors of the Al-Fe-Ni-Sc-Zr alloy, and the microstructure–mechanical property relationship was analytically discussed.

## 2. Materials and Methods

### 2.1. Materials Fabrication

The Al-1.75wt%Fe-1.25wt%Ni eutectic alloy is called an AlFeNi alloy hereafter for brevity. Its cast-ability, microstructure, mechanical properties, and thermal stability were reported in several former studies [17,18,45]. In this work, the micro-alloyed elements of 0.2wt%Sc and 0.2wt%Zr were co-added into this eutectic alloy, and thus the related microstructural features and mechanical performance were studied.

Experimental alloys were prepared using the common gravity casting method. In Table 1, both alloys were prepared according to the stoichiometric ratio, and the total weight of raw materials used in each casting process was 5 kg. For example, the AlFeNi-0.2Sc-0.2Zr alloy was melted in an iron crucible located inside the resistance furnace. Initially, the iron crucible was cleaned and covered with refractory paint to prevent excessive pollution of the melted alloy from crucible. Then, putting high-purity Al, Al-10wt%Ni, Al-20wt%Fe, Al-10wt%Zr binary alloys into the crucible, the melt was heated until 850 °C, and kept for 10 min. Subsequently, Al-2wt%Sc alloy was put in the melt. Then, a graphite rod was used to stir the melt, promoting the completed melting of various raw materials. Afterwards, the melt was refined and degassed for 10 min in a vacuum furnace preheated to 760 °C. When the melt was at 740 ± 5 °C, it could be cast into an iron mold. Subsequently, the casting was removed quickly from the mold and cooled in the air. The elemental compositions of both alloys were determined by the inductively coupled plasma atomic emission spectrometry (ICP-AES, iCAP7600, U.S.), as exhibited in Table 1.

### 2.2. The Heat Treatment Procedure

The Nabertherm furnace was used for heat treatment on alloy samples, and its maximum temperature was 680 °C. For the homogenization treatment, the alloy samples were held at 640 °C for 12 h or 24 h and quickly quenched in 25 °C water. For the aging treatment, the alloy samples were held at 300/350/400 °C for 0–12 h and cooled in the air.

### 2.3. Microstructure Characterization and Mechanical Testing

The microstructure was analyzed by optical microscopy (OM, ZEISS Axio Imager, Germany), scanning electron microscopy (SEM, TESCAN, Czech), energy-dispersive X-ray spectroscopy (EDX, U.K.), and Electron Back-Scattered Diffraction (EBSD, U.K.). The samples were grinded by emery papers from 400# to 2500# and polished by the diamond suspension. Furthermore, Image J (ProPlus 6.0) software was adopted to proceed with image analysis to determine the average grain size (*d*). Afterwards, the average grain size was defined as the diameter of a circle with the same area (*S*). This was calculated by $d=\sqrt{4S/\pi }$. In addition, The X-Ray Diffraction (XRD, D8 ADVANCE Da Vinci, U.S.) was used to detect the phase composition, and the specific condition included 2θ from 10° to 90° at a rate of 2 °/min.

The electrical conductivity test was used to evaluate the precipitation behaviors of the alloys, and it was performed with an electrical conductivity unit (Sigma2008A, China). The cylindrical samples have a diameter of 10 mm and a height of 7 mm, and the samples were grinded by emery papers from 400# to 2500# before testing. To derive the average value, there were five measuring times on each sample. These things considered, the Vickers hardness (HV) test was used to evaluate mechanical performance of the alloys. The hardness measurement was carried out on a hardness test machine (Carat 930, Germany) with a load of 98 N and 15 s dwelling time. In each sample, there were 5 points tested to achieve the average result. Furthermore, the tensile properties of the selected alloy were tested on a Zwick/Roell Z100 machine (Germany). The strain rate was 1 × 10^−3^ s^−1^, as controlled by the extensometer. The sample was made according to ASTM E8/E8M-21 (U.S.). For each alloy, there were six samples tested to achieve the average value.

## 3. Results

### 3.1. Microstructure and Performance of As-Cast Alloys

#### 3.1.1. Microstructure Characterizations of As-Cast Alloys

**Figure 1:**
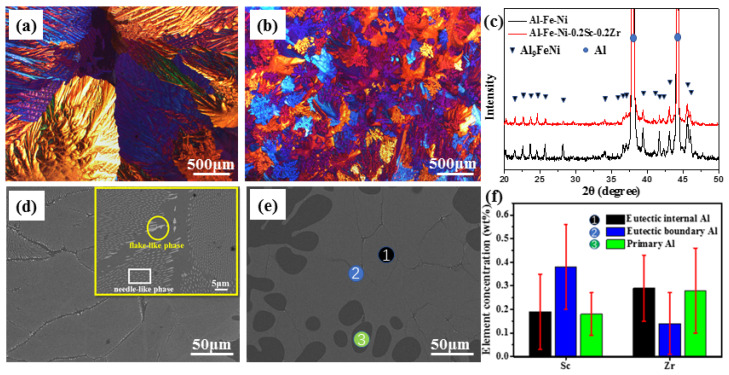
Microstructure features of the studied alloys including macro-grain structures: (**a**) AlFeNi; (**b**) AlFeNi-0.2Sc-0.2Zr. (**c**) XRD patterns of both alloys. Microstructures of as-cast alloys: (**d**) AlFeNi; (**e**) AlFeNi-0.2Sc-0.2Zr. (**f**) Both Sc and Zr contents detected by EDX at different positions in as-cast AlFeNi-0.2Sc-0.2Zr alloy.

The OM images of both AlFeNi (Figure 1a) and AlFeNi-0.2Sc-0.2Zr (Figure 1b) alloys are exhibited to describe their macro-grain structures. For the eutectic AlFeNi alloy (Figure 1a), it shows the very coarse grains with the average grain size of 2240 ± 300 μm. With the co-addition of Sc and Zr elements, there is comprehensive grain refinement of the AlFeNi-0.2Sc-0.2Zr alloys, where the average grain size is greatly reduced to 68 ± 54 μm. Normally, the decrease in alloy grain size and the increase in the primary Al area are mainly due to the heterogeneous nucleation induced by the primary Al_3_(Sc, Zr) phase. The similar phenomenon has been identified in Al-Sc-Zr-Si-Er [46] and eutectic Al-Fe-Ni-Sc-Zr alloys [47]. Liu et al. [48] also discussed the grain refinement and the related mechanism in Al-Zn-Mg-Cu alloys by Sc and Zr co-addition.

The XRD patterns of both as-cast alloys are presented in Figure 1c. For the as-cast AlFeNi alloy, its XRD pattern can be indexed to the α-Al (JCPDS #04-0787) and Al_9_FeNi phases ([49]). For the AlFeNi-0.2Sc-0.2Zr alloy, there are rarely new diffraction peaks for other phases. The co-addition of Sc and Zr elements does not change the main alloy phases. Figure 1d and e show the microstructures of both studied alloys. For the as-cast AlFeNi alloy, its general microstructure contains eutectic phase and eutectic-phase-free areas (EPFAs) [18], which can be seen clearly in the high-magnification image shown in the insert of Figure 1d. The shape of the eutectic phase located inside the grains is needle-like, and the shape of the eutectic phase located at the edge of grain boundary is flake-like. The needle-like or flake-like eutectic phases are both Al_9_FeNi as confirmed in Figure 1c, which is consistent with former research [18]. The formation process and microstructure feature of the eutectic phase in this eutectic alloy can be referred to in our previous report [17,18].

Once the eutectic alloy is micro-alloyed by Sc and Zr elements, the microstructure change in the AlFeNi-0.2Sc-0.2Zr alloy is presented (Figure 1e). Generally, the eutectic structure has been influenced by the Sc and Zr co-addition to a small extent. The details of the eutectic structure are similar to those of the AlFeNi alloy. The primary Al region in the AlFeNi-0.2Sc-0.2Zr alloy has increased significantly with the co-addition of alloying elements. Normally, the Al matrix can be divided into three regions: eutectic internal Al (Mark 1), eutectic boundary Al (Mark 2), and primary Al (Mark 3), as shown in Figure 1e.

The elements in the as-cast AlFeNi-0.2Sc-0.2Zr alloy are not uniformly distributed in the matrix. It was reported that Sc is more likely to accumulate in inter-dendritic regions, and Zr is more likely to accumulate in dendritic regions [33,41]. This is because Sc undergoes a eutectic reaction during solidification, and Zr undergoes a peritectic reaction. Due to the different solidification process, Sc is pushed to the front of the interface during the solidification process and enriched in the final solidified part. Comparably, Zr is inclined to be enriched in the nucleated matrix during the solidification process. This behavior was discussed in both Al-Sc-(Zr) alloys [41,44] and Al-Fe-Ni-Sc-(Zr) alloys [45,47].

In Figure 1f, both Sc and Zr contents are shown, as detected by EDX at three marking positions in the as-cast alloy. Clearly, the Sc content reaches 0.37wt% in eutectic boundary Al (Figure 1f), which is almost twice the added Sc content (Table 1). Herein, the Sc contents in both primary Al and eutectic internal Al are lower than the added Sc content. Comparably, the distribution tendency of Zr element is opposite to the Sc element. It can be found that the Zr content in eutectic boundary Al is 0.13wt%, which is much less than the added Zr amount. The Zr contents in the other two regions exceed the added amount and are twice over the eutectic boundary Al. During the solidification process, both Sc and Zr should not dissolve in the eutectic phase but only dissolve in the Al matrix. In the Al-Fe-Ni-Sc-(Zr) system, both Sc and Zr elements are stimulated by the eutectic phase to be more unevenly distributed than Al-Sc-(Zr) dilute alloys [33,41,45,47].

#### 3.1.2. Influence of Aging Method on Hardness and Electrical Conductivity of As-Cast AlFeNi-0.2Sc-0.2Zr Alloy

**Figure 2:**
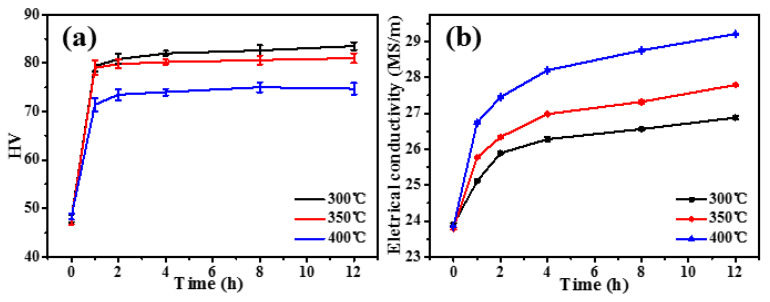
Variation of (**a**) Vickers hardness and (**b**) electrical conductivity during single-step aging at 300 °C, 350 °C, and 400 °C for AlFeNi-0.2Sc-0.2Zr alloy.

The Vickers hardness (Figure 2a) and electrical conductivity (Figure 2b) are exhibited for the isothermal aging at 300 °C, 350 °C, and 400 °C on the as-cast AlFeNi-0.2Sc-0.2Zr alloy. In each temperature, the hardness of each alloy increases rapidly in the first 4 h (Figure 2a), and the electrical conductivity shows a similar trend (Figure 2b). This means that a large amount of solutes (Sc and Zr) are precipitated to form Al_3_(Sc, Zr) during this process. As the aging continues, the hardness values remain unchanged at each temperature for the alloys, but the electrical conductivities increase slightly.

In Figure 2a, the peak-aged hardness decreases from ~84 HV to ~73 HV with the aging temperature rising from 300 °C to 400 °C for the as-cast AlFeNi-0.2Sc-0.2Zr alloy. With the increasing aging temperature, the reduced peak-aged hardness is mainly due to the change in the strengthening mechanisms of the precipitate-enhanced alloy. Two involved strengthening mechanisms include (i) a shearing mechanism and (ii) a dislocation bypass mechanism [26,28]. The room-temperature enhancement of precipitate-strengthened Al-Sc-Zr alloys can be explained separately or in combination with both mechanisms. When the radius of the precipitate is small (r < 1.5–2 nm) [26], dislocations can directly cut through the precipitate, and the shear mechanism can be used to explain the strengthening effect. Within the scope of the shear mechanism, as the precipitate size increases, the enhancement effect is improved [26]. When the precipitate radius grows larger, it is difficult for dislocations to cut directly through the precipitate. Thus, the dislocation loops form around the precipitate, and the dislocation bypass mechanism occurs. In this manner, the strengthening effect should decrease with the rising size of the precipitate [24,26]. Typically, the precipitate radius of around 2–3 nm has the superior strengthening effect [24,26,46]. With the elevated aging temperature, the critical nucleation radius of the precipitate increases [30,37,50]. At the higher aging temperatures, the precipitate radius must be greater than 2 nm, and the precipitate strengthening mechanism is a bypass mechanism at this time [26]. Therefore, as the temperature increases, the increment of aging strengthening decreases.

The hardness variation during the aging process corresponds to the change in the electrical conductivity. In Figure 2b, the alloy presents two features in the electrical conductivity. The first feature is that the electrical conductivity increases with the aging temperature, and the second feature is that the enhancement of electrical conductivity between 400 °C and 350 °C is larger than that between 350 °C and 300 °C. Thus, both features should be mainly because Zr owns the slower diffusion rate over Sc. At the same temperature, Zr has a two orders of magnitude lower diffusion rate than that of Sc [16,24]. Therefore, the low conductivity value indicates that a large amount of Zr is still not completely precipitated at 300 °C and 350 °C. Therefore, the precipitation of Zr continues as the aging progresses, and the electrical conductivity has a slow continuous rise. At 400 °C, the diffusion rate of Zr is increased, and the precipitation behavior is more pronounced. Finally, the enhancement of the electrical conductivity is larger between 400 °C and 350 °C compared to that between 350 °C and 300 °C.

It is known that Zr is the main factor that enables Al_3_(Sc, Zr) to withstand a high temperature of 400 °C [33,41]. In the as-cast alloy, the distribution behaviors of both Sc and Zr are uneven. Therefore, the precipitates in the Sc-gathered grain boundary cannot effectively form a core–shell structure due to the lack of Zr during the aging process. Then, they should coarsen rapidly at higher temperatures, resulting in a lower precipitation response of single-step aging at both 350 °C and 400 °C. This means that the alloy containing both Sc and Zr elements should not be suitable for the single-step aging treatment at temperatures exceeding 300 °C. However, the complete precipitation for the solutionized atoms is inadequate at 300 °C, especially for Zr atoms [33,41].

According to the above analysis, there are two problems in the single-step aging at a determined temperature. Firstly, Sc and Zr have large differences in the diffusion rate [24]. As a result, Zr cannot be completely precipitated at lower temperature, and the Sc precipitated at a higher temperature should rapidly coarsen to lose the strengthening effect. Secondly, Sc and Zr are unevenly distributed in the matrix. Therefore, the precipitates cannot form a stable core–shell structure effectively and are easier to coarsen at the grain boundary, due to the lack of Zr. Overall, it is necessary to optimize the aging procedures to improve the aging response of the as-cast AlFeNi-0.2Sc-0.2Zr alloy.

**Figure 3:**
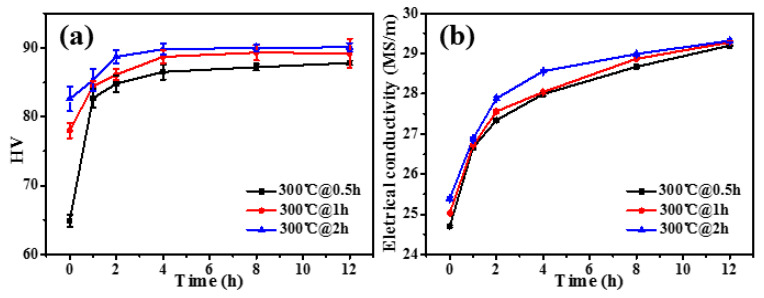
Evolution of (**a**) Vickers hardness and (**b**) electrical conductivity during the second-step aging treatment (isothermal aging at 400 °C previously aged 0.5 h, 1 h, and 2 h at 300 °C) for as-cast AlFeNi-0.2Sc-0.2Zr alloy.

Based on these considerations, a two-step aging treatment is performed: (i) A short-time aging procedure at a lower temperature is carried out as the first stage to maximize the number density of the precipitates. (ii) A long-time aging procedure at a higher temperature is performed as the second stage to optimize the nanostructure of the precipitate, making the Zr completely precipitate during this aging process. Seidman et al. [24] used the root-mean-square (RMS) diffusion distance ($\sqrt{4Dt}$) to evaluate the element diffusion in the matrix [24]. Through the calculation and experimental verification, it is determined that 300 °C is good for Sc precipitation in the first-step aging procedure, and 400 °C is advantageous for Zr precipitation in the second-step aging procedure. In detail, the as-cast AlFeNi-0.2Sc-0.2Zr alloy is aged from 0.5 to 2 h at 300 °C for the first-step aging duration. Then, the second-step aging procedure is performed at 400 °C from 0 to 12 h to show the corresponding aging response by both Vickers hardness (Figure 3a) and electrical conductivity tests (Figure 3b) on the alloy.

In Figure 3a, the hardness values at the starting point are obviously different, which should be caused by the different size and number density of precipitates influenced by the different time of the first-step aging treatment. In the following aging treatment, the as-cast alloy is hardened rapidly in the beginning 2 h, and the alloy basically reaches the peak-aged state after 4 h. This tendency is independent of the aging duration of the first-step aging treatment. Once the alloy reaches the peak-aged state, the hardness can remain unchanged for a long duration at 400 °C. Such a feature has complied well with the thermal stability for reported Al-Sc-Zr materials [24,41,47]. In Figure 3b, the electrical conductivity shows a similar change trend for the as-cast alloy that experienced the second-step aging treatment. Herein, the alloy can reach a close value irrespective of the aging condition. This means that during the second-step aging process, Zr should make a substantial contribution to create precipitates with elevated thermal stability. Generally, the as-cast AlFeNi-0.2Sc-0.2Zr alloy has exhibited the optimum aging response by the two-step aging treatment for 300 °C@2 h + 400 °C@4 h (Figure 3a). The corresponding hardness is ~91 HV, which is much higher than that of ~84 HV obtained by single-step aging treatment (Figure 2a) for the alloy.

**Figure 4:**
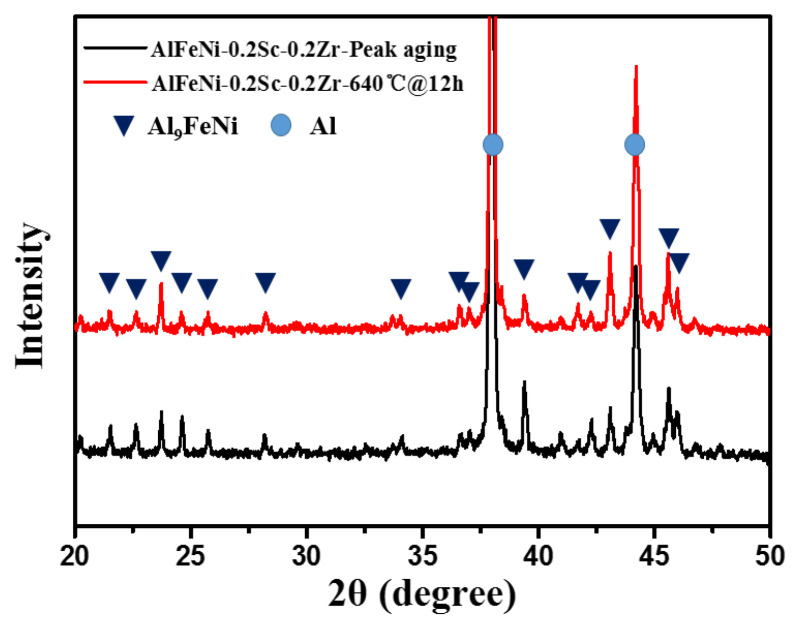
XRD patterns of AlFeNi-0.2Sc-0.2Zr alloys with different heat treatment.

These things considered, the phase analyses of peak-aged alloys are also conducted by XRD, as shown in Figure 4. It is found that there is rarely any change among the phases in the alloy, where Al and Al_9_FeNi phases still dominate. In fact, the Al_3_(Sc, Zr) nano-precipitated phases appear in the Al matrix of peak-aged alloys, but these phases are too small to be detected by XRD. Normally, these nano-precipitated phase needs to be observed by SEM or TEM [26].

### 3.2. Microstructure and Performance of As-Homogenized AlFeNi-0.2Sc-0.2Zr Alloys

For the alloy containing both the alloying elements Sc and Zr, there are two issues in the precipitation process. The first issue is the unsynchronized precipitation of elements. Two-step aging is designed to solve the problem of unsynchronized element precipitation, which can greatly improve the aging response. The second issue is the uneven distribution of both Sc and Zr. To solve such a problem, homogenization treatment before the aging treatment is applied in this following section.

#### 3.2.1. Microstructure Characterizations of As-Cast Alloys

**Figure 5:**
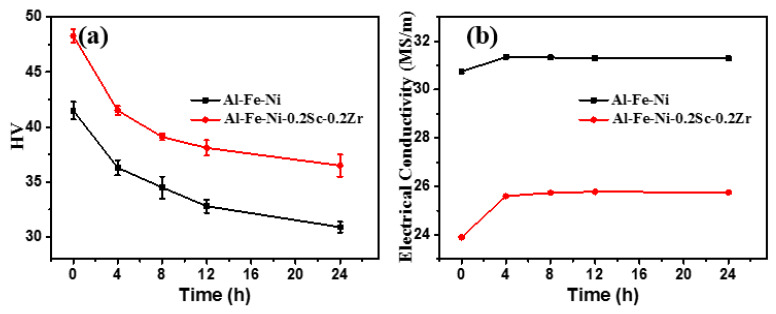
Evolution of (**a**) Vickers hardness and (**b**) electrical conductivity of experiment alloys during homogenization treatment at 640 °C.

According to the previous study, we chose 640 °C as the homogenization temperature [43,44]. The hardness values (Figure 5a) for both as-cast AlFeNi and AlFeNi-0.2Sc-0.2Zr alloys are greatly reduced during homogenization treatment. Within the first 8 h, both alloys show decreased hardness of 7–9 HV. Probably, the decreased hardness is related to the microstructure evolution of the eutectic phase. Bian et al. [17] reported that the Al_9_FeNi phase stayed stable at a temperature as high as 400 °C, but it experienced the fully coarsening stage under 640 °C. In the first few hours, the needle-like eutectic phase is broken and spheroidized, and the hardness decreases rapidly. The spheroidized eutectic phase can continue to coarsen during the heat exposure process, and the hardness value should slowly decrease during this process. As the homogenization proceeds, the rate of the hardness decrease slows down, indicating that the coarsening rate of the eutectic structure gradually decreases (Figure 5a).

During homogenization treatment, either defect evolution or primary precipitates dissolved in the matrix can be reflected by the variation in electrical conductivity [44]. Either the elimination of defects or the precipitation of solute atoms can increase the alloy’s electrical conductivity. The decrease in electrical conductivity represents an increase in the amount of solutionized atoms in the alloy. The dissolution of some primary phases should account for this effect. [44]. In Figure 5b, the electrical conductivity of the AlFeNi alloy rises slightly during this homogenization. Then, the increment is 0.55 MS/m. This may be caused by the elimination of defects (i.e., vacancies or dislocations) in the alloy [51]. However, the electrical conductivity of the AlFeNi-0.2Sc-0.2Zr alloy has a larger increase after the homogenization process, and the increasing value reaches 1.88 MS/m. As the homogenization period extends, the conductivity ever varies. This means that after eliminating the influence of defects on conductivity, it can be acknowledged that the solutionized atoms in the matrix are reduced.

**Figure 6:**
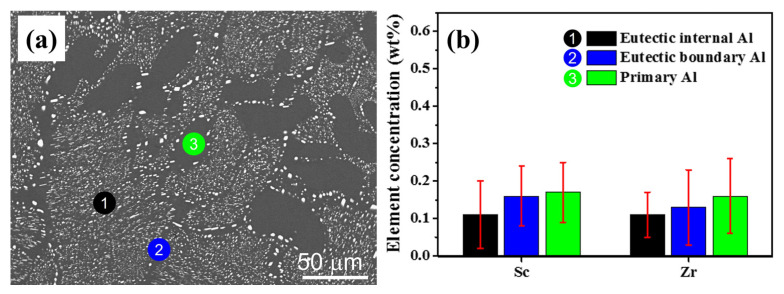
As-homogenized AlFeNi-0.2Sc-0.2Zr alloy: (**a**) microstructures; (**b**) both Sc and Zr contents detected by EDX at different positions. Both Sc and Zr contents detected by EDX at different positions in as-homogenized AlFeNi-0.2Sc-0.2Zr alloy.

For the typical microstructures of as-homogenized AlFeNi-0.2Sc-0.2Zr alloy (Figure 6a), the alloying element distribution is relatively uniform after 12 h homogenization treatment, as shown by the EDX result (Figure 6b). Notably, the element contents detected at corresponding positions in the matrix are greatly reduced over the as-cast alloy (Figure 1f). It is also in line with the findings obtained from Figure 5b.

#### 3.2.2. Microstructures of As-Homogenized AlFeNi-0.2Sc-0.2Zr Alloys

**Figure 7:**
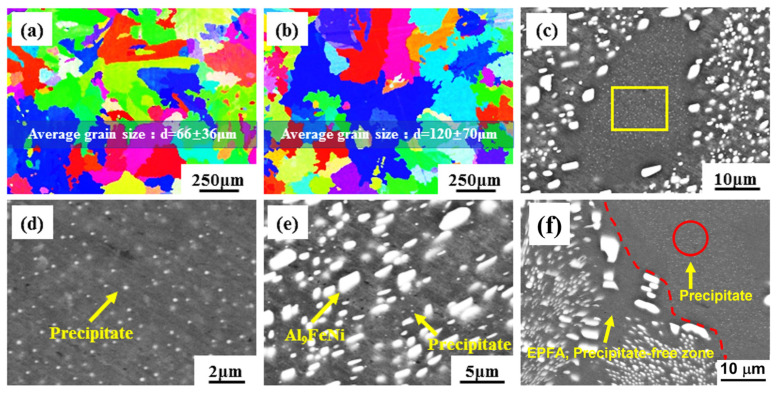
EBSD patterns of AlFeNi-0.2Sc-0.2Zr alloy in (**a**) as-cast and (**b**) as-homogenized states. Microstructure of as-homogenized AlFeNi-0.2Sc-0.2Zr alloy: (**c**,**e**,**f**) are primary Al, eutectic phase, and EPFA, respectively; (**d**) is the enlarged view of the yellow rectangle region in (**c**).

After 640 °C homogenization, the alloy microstructure changed significantly. Therefore, the as-homogenized alloy was further phase-identified using XRD. Generally, the eutectic Al and Al_9_FeNi phases remained in the alloy (Figure 4). Furthermore, Figure 7a,b show the grain structures of the AlFeNi-0.2Sc-0.2Zr alloy in the as-cast and as-homogenized states, respectively. For the as-cast state, it has an average grain size of 66 ± 36 μm. After the 12 h homogenization treatment, the alloy has a larger average grain size (120 ± 70 μm), which almost doubles over the as-cast state. Additionally, the eutectic phase also changed its morphology. In Figure 7e, the needle-like eutectic phase in the as-cast alloy undergoes comprehensive spheroidization and coarsening during the homogenization process. The average diameter distribution of the eutectic phase after coarsening is 1~5 μm. The collapse of the eutectic framework may cause a negative impact on the high temperature mechanical properties of the alloy [17,52].

There are many spherical precipitates in an as-homogenized AlFeNi-0.2Sc-0.2Zr alloy, which are ever found in the as-cast alloy. Figure 7c shows a typical primary Al area, and a lot of fine precipitates can be seen evenly distributed in this area. The diameters of these spherical precipitates are ~100–200 nm, as shown in the magnified image (Figure 7d). When it comes to the eutectic Al revealed in Figure 7e, many small precipitates similar to those in primary Al can be seen. For the eutectic internal Al, it has a much smaller number density of precipitates than that of the primary Al. However, as the EPFA marked in Figure 7f, precipitates can rarely be seen in this area.

**Figure 8:**
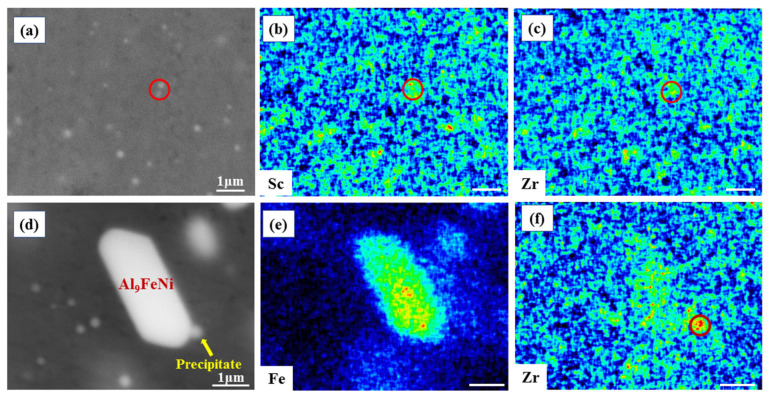
EDX analysis of precipitation in the AlFeNi-0.2Sc-0.2Zr alloy after 12 h homogenization at 640 °C: (**a**–**c**) are obtained in primary Al; (**d**–**f**) are obtained around the eutectic phase. (The scale bars are all 1 μm).

After homogenization treatment, the composition of the newly discovered precipitates in the matrix can be roughly determined by EDX. Figure 8a shows the precipitates in the primary Al area, and the corresponding distributions of Sc and Zr are exhibited in Figure 8b,c, accordingly. Clearly, these phases are rich in Sc and Zr elements, which are supposed to be Al_3_(Sc, Zr). In Al, Zr has a solubility of ~0.226wt% at 640 °C from the Al-Zr phase diagram [53]. Meanwhile, there is no precipitation in the EPFA where the Zr content is relatively low. It can be speculated that the appearance of the precipitates may be mainly caused by the incomplete solution of Zr. When the Zr element is precipitated during the homogenization process, a part of the Sc element should also participate in precipitation. Therefore, the element content in the matrix should be reduced, which is detrimental to precipitation strengthening.

Figure 8d shows the element mapping analyses around the eutectic phase. Herein, the larger phase is the coarsened Al_9_FeNi, and the smaller phase is Al_3_(Sc, Zr) precipitated during the homogenization process. Clearly, Al_3_(Sc, Zr) precipitates form along the interface, where the elements segregation occurs. In Figure 8f, the Zr elements are more concentrated around the eutectic phase. This can explain why less Al_3_(Sc, Zr) phases are inside eutectic Al than the primary Al (Figure 7e).

#### 3.2.3. Hardness and Electrical Conductivity of As-Homogenized AlFeNi-0.2Sc-0.2Zr Alloy

**Figure 9:**
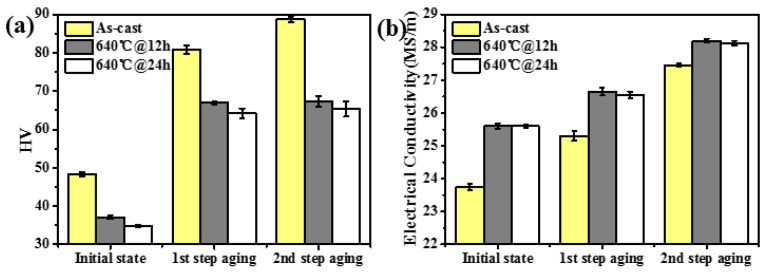
Evolution of (**a**) Vickers hardness and (**b**) electrical conductivity of as-homogenized AlFeNi-0.2Sc-0.2Zr alloy during aging.

In the previous section, the optimized two-step aging method for this eutectic alloy was decided. The same method is applied for the homogenized alloy, and the electrical conductivity and hardness of this process are exhibited in Figure 9. The initial state refers to the as-cast state, which is either 640 °C@12 h or 640 °C@24 h (Figure 9). For the two-step peak-aged treatment, the first-step aging is 300 °C@2 h, and second-step aging is 400 °C@4 h.

The hardness increment of the homogenized alloy is ~30 HV which is greatly reduced compared to that of ~42 HV of the as-cast state. This is because part of the Sc and Zr elements form precipitates with the size of 100~200 nm during homogenization (Figure 7d), of which the strengthening effect is slight. Figure 9a shows the alloy hardness after 12 h and 24 h homogenization treatment at 640 °C. Although the overall hardness of the latter treatment is lower, the hardness increases in both treatments are almost similar. Furthermore, the electrical conductivity of the alloy hardly changes for both treatments. This suggests that the combined Sc and Zr elements in the alloy have a certain limited solubility in current homogenization treatment.

## 4. Discussion

The above analysis suggests that homogenization treatment is not suitable for AlFeNi-0.2Sc-0.2Zr. This is because the micro-alloyed elements exceed the solutionized limit in the AlFeNi-0.2Sc-0.2Zr alloy. Therefore, the Al_3_(Sc, Zr) phase should precipitate during the homogenization process, and then the practical solute contents for both Sc and Zr elements remaining in the Al matrix should be reduced. Since there are inadequate solute elements dissolved in the matrix, the precipitated particles during the aging process are incapable of inducing an intensive strengthening effect on the Al matrix. During the homogenization process, the Zr exceeding the solid solubility entrains the Sc element to form a precipitated phase, which causes the atomic concentration in the matrix to decrease. It is necessary to determine both the Sc and Zr contents which can be redissolved into Al matrix through the homogenization process.

The peak-aged hardness induced by the two-step aging treatment is used to determine the alloy composition suitable for the homogenization treatment in these AlFeNi-Sc-Zr alloys. There are two prerequisites in this process: (1) The alloy that experienced the homogenization procedure can show a certain aging response. This can provide indirect proof of the maximum element contents through the heat treatment and hardness experiments. (2) Zr can be dissolved and its solid solubility is higher than 0.2wt% in the Al matrix [53]. Theoretically, it can be completely dissolved during the homogenization process. However, Zr elements should accumulate in the primary Al and eutectic Al regions due to element segregation (Figure 1f). The precipitates (Figure 7c,e) observed in the current experiment may be due to the Zr concentration exceeding 0.2wt% in these regions (Figure 1f). The phenomenon of local precipitation due to Zr segregation during the homogenization process has also been mentioned in Kang’s work on the Al-0.06at%Sc-0.06at%Zr-0.008at%Er alloy [44]. They also found that by reducing the Zr content, an Al-0.06at%Sc-0.03at%Zr-0.008at%Er alloy was obtained, and the related mechanical performance was improved via homogenization treatment [44].

**Figure 10:**
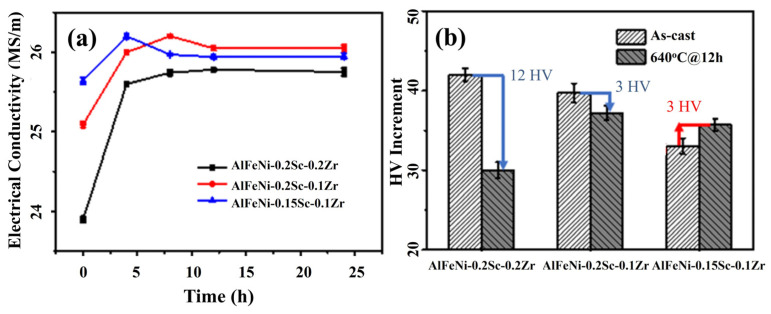
(**a**) Electrical Conductivity changes in three alloys in the process of homogenization; (**b**) peak-aged hardness increment of as-cast and as-homogenized alloys.

Therefore, based on the AlFeNi-0.2Sc-0.2Zr alloy, the Zr content was reduced to 0.1wt% to obtain the AlFeNi-0.2Sc-0.1Zr alloy by the same casting method. In Figure 10a, the electrical conductivity of AlFeNi-0.2Sc-0.1Zr shows a small increase after homogenization treatment. This represents how some of the elements precipitated during the homogenization. Further hardness testing is conducted to confirm the change in solubility. First, once the Zr content is decreased, the peak-aged hardness increment of the as-homogenized AlFeNi-0.2Sc-0.1Zr alloy reaches ~36 HV, which is higher than that of ~30 HV of as-homogenized AlFeNi-0.2Sc-0.2Zr (Figure 10b). Therefore, the reduced Zr content offers a positive impact on homogenization treatment. However, the increase in the peak-aged hardness of the as-homogenized AlFeNi-0.2Sc-0.1Zr alloy is reduced by ~3 HV over its as-cast state (Figure 10b). The decrease in hardness suggests that the solutionized element is still incomplete under this component.

Under the premise of the Zr content reduction, it is reasonable to suspect that the excessive Sc element can also cause precipitation and even coarsening during the homogenization process. In order to verify this conjecture, we prepared the as-cast AlFeNi-0.15Sc-0.1Zr alloy, which was designed to further reduce the content of total solutionized atoms in comparison with that of AlFeNi-0.2Sc-0.1Zr. In Figure 10a, the electrical conductivity has a very slight rise during the homogenization process. This conductivity change is similar to that of AlFeNi at 640 °C. It can be considered that this is due to the annihilation of defects [51]. This shows that there is rare reduction of the solutionized elements in the matrix. From the hardness change (Figure 10b), the peak-aged hardness increment of the as-homogenized AlFeNi-0.15Sc-0.1Zr alloy elevated by ~3 HV over its as-cast alloy state. Although the degree of increase is small, it also shows that the alloy of this composition is suitable for homogenization.

Tensile experiments at room temperature were made on both AlFeNi-0.2Sc-0.2Zr and AlFeNi-0.15Sc-0.1Zr alloys at as-cast + peak-aging state and as-homogenized (12 h @640 °C) + peak-aging state. The results of the yield strength (YS), ultimate tensile strength (UTS), and elongation (EL%) are listed in Table 2. Compared to hardness, tensile properties can reflect the evolution of mechanical properties more comprehensively. In Table 2, the elongations of both alloys increase significantly after homogenization. It is not difficult to see that the heat treatment schedule is crucial to control alloy properties. Thus, the alloy composition matching the heat treatment schedule needs to be carefully identified. Specifically, both the YS and UTS valies of AlFeNi-0.2Sc-0.2Zr reduce significantly, which are consistent with the variation trend of hardness. Obviously, Sr and Zr co-precipitation during homogenization is the main reason. In comparison, the UTS value of the AlFeNi-0.15Sc-0.1Zr alloy decreases a little, but the YS value remains almost unchanged. Since the rod-like eutectic phase should spheroidize during homogenization, the mechanical performance of the AlFeNi matrix should be reduced to a certain extent. That is to say, the unchanged yield strength can be attributed to the increment caused by the more uniform distribution of Sc and Zr after homogenization. On the other hand, it is suspected that the reduction in the UTS value is derived from the decline of the work-hardening capacity caused by the spheroidization of the rod eutectic phase.

Above all, a proper heat treatment schedule can achieve the purpose of regulating the mechanical properties of the AlFeNi-Sc-Zr alloys. Meanwhile, the additional amount of co-micro-alloyed elements can also affect the effect of heat treatment. Although more Sc + Zr addition in the AlFeNi-0.2Sc-0.2Zr alloy can induce a better strengthening effect in the as-cast and peak-aging treatment, they also lead to the failed homogenization process. At the same time, Bian et al. [47] found adding too much Sc and Zr addition should reduce the alloy grain size. This should be detrimental to the alloy high-temperature performance. Furthermore, the reduced Sc + Zr addition in the AlFeNi-0.15Sc-0.1Zr alloy can be adapted to a wider range of heat treatment schedule to meet the performance requirements of alloys under different conditions. Meanwhile, the reduced micro-alloying elements can reduce the cost, which is practically beneficial for industrial application.

## 5. Conclusions

This study investigated the Al-Fe-Ni eutectic alloy micro-alloyed by Sc and Zr elements. Through Vickers hardness and electrical conductivity experiments, the precipitation behavior of Sc and Zr manipulated by the heat treatment was studied. The following conclusions can be drawn as follows:

(1) When the single-step aging treatment was carried out on the AlFeNi-0.2Sc-0.2Zr alloy, the aging response was improved with the reduced aging temperature. In this manner, 300 °C single-step aging can obtain the aging increment of ~36 HV.
(2) In order to minimize the diffusion difference between Sc and Zr elements, the two-step aging method showed better results than those of single-step aging for the AlFeNi-0.2Sc-0.2Zr alloy. In detail, this alloy obtained the highest peak-aged hardness increase of ~42 HV by the two-step aging condition of 300 °C@ 2 h + 400 °C@ 4 h.
(3) Since there was uneven distribution of both Sc and Zr alloying elements, the homogenization treatment for the alloy was discussed. Although the solid solubility of the Zr element was greater than 0.2wt% at 640 °C, the AlFeNi-0.2Sc-0.2Zr alloy was not suitable for homogenization treatment due to the precipitation of Al_3_(Sc, Zr) during homogenization influenced by element segregation.

Therefore, both Sc and Zr contents were reduced simultaneously, and the aging response of AlFeNi-0.15Sc-0.1Zr alloys was improved after homogenization treatment. The underlying mechanisms for this alloy design and the corresponding microstructure–mechanical property relationship were analytically discussed.

## Figures and Tables

**Table 1:** Chemical compositions of AlFeNi-Sc-Zr alloys (wt%).

Alloy		Fe	Ni	Sc	Zr
AlFeNi	* Nom. Comp.	1.75	1.25	-	-
	ICP results	1.67 ± 0.02	1.25 ± 0.015		
AlFeNi- 0.2Sc-0.2Zr	Nom. Comp.	1.75	1.25	0.2	0.2
	ICP results	1.66 ± 0.012	1.22 ± 0.03	0.19 ± 0.005	0.2 ± 0.002

* Nom. Comp.: nominal compositions.

**Table 2:** Tensile properties of both alloys at as-cast + peak-aging state and as-homogenized (12 h @640 °C) + peak-aging state.

Alloy		YS/MPa	UTS/MPa	EL/%
AlFeNi- 0.2Sc-0.2Zr	as-cast + peak-aging	240 ± 3	297 ± 4.4	12.9 ± 0.3
	as-homogenized + peak-aging	156 ± 0.9	206 ± 2.8	22.4 ± 0.6
AlFeNi- 0.15Sc-0.1Zr	as-cast + peak-aging	165 ± 2.5	237 ± 5	11.2 ± 0.8
	as-homogenized + peak-aging	164 ± 3	223 ± 3.1	24.4 ± 0.5

## Data Availability

Data are contained within the article.
